# Association between red blood cell distribution width to albumin ratio and prognosis in patients with sepsis-associated acute kidney injury: a retrospective cohort study

**DOI:** 10.3389/fmed.2026.1724095

**Published:** 2026-02-03

**Authors:** Keke Wei, Huiquan Huang, Zheng Su, Haili Zeng, Jinqiu Cen, Hui Li

**Affiliations:** 1 Department of Critical Care Medicine (Ward 1), Baise People's Hospital, Affiliated Southwest Hospital of Youjiang Medical University for Nationalities, Youjiang District, Baise, Guangxi, China; 2 Emergency Intensive Care Unit, Baise People's Hospital, Affiliated Southwest Hospital of Youjiang Medical University for Nationalities, Youjiang District, Baise, Guangxi, China

**Keywords:** clinical outcomes, cohort study, LAR ratio, RAR ratio, SA-AKI

## Abstract

**Background:**

The red blood cell distribution width-to-albumin ratio (RAR) has shown prognostic value in sepsis, diabetes comorbidities, and cancer (CA). However, its relevance to clinical outcomes in sepsis-associated acute kidney injury (SA-AKI) remains unclear. This study aimed to explore this relationship.

**Methods:**

A retrospective cohort study was conducted on SA-AKI patients admitted to the Intensive Care Unit of Baise People's Hospital, Guangxi, from May 2022 to May 2025. The primary endpoint was 28-day all-cause mortality. The prognostic utility of RAR was assessed using multivariable Cox regression, restricted cubic splines (RCS), Kaplan–Meier survival curves with log-rank tests, stratified analysis, receiver operating characteristic (ROC) curves, subgroup, and sensitivity analyses.

**Results:**

Among the 161 enrolled patients (mean age 63.7 ± 16.6 years), the mean RAR was 6.95 ± 2.81%/g/dl. A linear association between RAR and mortality was observed (non-linearity *p* = 0.162). After multivariable adjustment, the highest RAR quartile (Q4: >8.31) was associated with a hazard ratio (HR) of 7.52 (95% CI: 2.24–25.29) compared to Q1 (< 5.07). Kaplan–Meier analysis revealed significantly higher mortality in the high-RAR groups (*p* < 0.001). The area under the ROC curve (AUC) for RAR in predicting 28-day mortality was 0.694 (95% CI: 0.612–0.776). Combining RAR with the lactate-to-albumin ratio (LAR) significantly enhanced predictive accuracy (AUC: 0.777; 95% CI: 0.703–0.851; *p* = 0.043 vs. RAR alone).

**Conclusion:**

Elevated RAR independently predicts adverse early prognosis in SA-AKI, with higher levels correlating with increased 28-day mortality. The combination of RAR and LAR significantly improves mortality prediction in this cohort.

## 1 Introduction

Sepsis-associated acute kidney injury (SA-AKI) is a common and critical complication in intensive care settings, associated with significant mortality risk. The systemic inflammatory response triggered by sepsis leads to renal dysfunction through various mechanisms, with an increasing incidence observed in recent years (1). SA-AKI contributes to substantial morbidity and mortality (2). Following the Sepsis-3.0 definition update in 2016, international multicenter studies consistently show that sepsis patients who develop AKI have a 2–3-fold higher hospital mortality risk (3). Accurate prognostication in SA-AKI is critical for enabling timely and aggressive interventions (4).

While established scoring systems are correlated with SA-AKI outcomes (5, 6), their clinical applicability is hindered by operational complexity, reducing their effectiveness as bedside predictive tools. Consequently, there is an unmet need for readily available biomarkers with strong predictive power to identify high-risk patients and guide therapeutic decision-making.

Red blood cell distribution width (RDW), a routine hematological measure of erythrocyte size variability (anisocytosis), is a cost-effective prognostic indicator across various conditions, including cardiovascular, renal, metabolic, and hepatic disorders (7). Emerging evidence highlights RDW as a marker of inflammatory load and oxidative stress, with prognostic significance in both sepsis and AKI populations (8, 9). Serum albumin, an acute-phase reactant, reflects nutritional status and exerts anti-inflammatory effects by reducing oxidative stress and preventing endothelial apoptosis (10, 11). Its prognostic value in sepsis is well-established (12).

The red cell distribution width-to-albumin ratio (RAR) is a novel inflammatory biomarker that integrates these pathways. Previous studies have demonstrated its prognostic value in conditions such as diabetic ketoacidosis (13), diabetic retinopathy (14), malignancies (15), and sepsis (16). However, the relationship between RAR and clinical outcomes, specifically in SA-AKI patients, remains unexplored.

This retrospective cohort study aims to assess the prognostic value of RAR in SA-AKI patients admitted to the intensive care unit of Baise People's Hospital, Guangxi, from May 2022 to May 2025.

## 2 Methods

### 2.1 Data source

This retrospective cohort study included SA-AKI patients admitted to the ICU of Baise People's Hospital, Guangxi, China, from May 2022 to May 2025. Ethical approval was granted by the Institutional Review Board (KY2022030401), with a waiver of informed consent due to the study's retrospective design. The study adhered to the Declaration of Helsinki and followed STROBE reporting guidelines.

### 2.2 Study population

Inclusion criteria: First-time hospital and ICU admission with SA-AKI.

Exclusion criteria: Age < 18 years; missing RDW or albumin data; pre-existing CKD; ICU stay < 24 h; human albumin infusion within 48 h prior to ICU admission; >10% missing covariate data.

The diagnosis of SA-AKI required fulfillment of dual criteria: Sepsis-3 guidelines for sepsis (17) and KDIGO standards for acute kidney injury (AKI) (18). Additionally, AKI manifestation must occur within 7 days following sepsis identification (any stage). Sepsis was characterized as life-threatening organ dysfunction resulting from a dysregulated host response to infection. This required evidence of confirmed or suspected infection during the initial 24-h ICU admission period, accompanied by a Sequential Organ Failure Assessment (SOFA) score ≥2. AKI was established through meeting any single criterion below: (1) serum creatinine elevation ≥0.3 mg/dl (≥26.5 μmol/L) over a 2-day period; (2) creatinine levels rising to ≥1.5times baseline values within the preceding week; or (3) sustained urine production ≤ 0.5 ml/kg/h for six or more hours.

### 2.3 Data collection

To minimize confounding from subsequent treatments, the first available measurements within 24 h of ICU admission were collected, including RDW, blood lactate, and serum albumin. Potential confounders included: demographic variables (age, gender), comorbidities [coronary artery disease (CAD), diabetes mellitus (DM), cerebrovascular accident (CVA), cancer (CA), high blood pressure (HBP)], clinical parameters [heart rate (HR), body temperature (T), mean arterial pressure (MAP), respiratory rate (RR), SOFA score, mechanical ventilation (MV), vasoactive agents, Acute Physiology and Chronic Health Evaluation II (APACHE II) score, continuous renal-replacement therapy (CRRT)], laboratory indices [Hematology: white blood cells (WBC), hematocrit (HCT), platelets (PLT), hemoglobin (HGB), red-cell distribution width (RDW); Biochemistry: albumin (ALB), potassium (K), sodium (Na), lactate (lac), PaO_2_/FiO_2_ ratio, blood urea nitrogen (BUN); Coagulation: prothrombin time (PT), D-dimer, activated partial thromboplastin time (APTT); Liver function: aspartate aminotransferase (AST)], alanine aminotransferase (ALT), Total Bilirubin (TBil), Direct Bilirubin (DBil), Indirect Bilirubin (IBil), and derived ratios (RAR: RDW/albumin; LAR: lactate/albumin).

Missing continuous data were imputed using multiple imputation by chained equations (5 imputations), with covariates showing >10% missingness excluded from the analysis. All parameters reflect the first measurements obtained within 24 h of ICU admission. The primary endpoint was 28-day all-cause mortality.

### 2.4 Statistical analysis

Stratification was based on RAR quartiles (Q1–Q4). Descriptive statistics were calculated as follows: continuous variables were expressed as mean ± *SD* or median (IQR), and comparisons were made using ANOVA or *t*-tests; categorical variables were reported as *n* (%) with comparisons performed using χ^2^ or Fisher's exact tests. The primary analysis involved multivariable Cox regression to assess the association between RAR and mortality, reporting hazard ratios (HRs) with 95% confidence intervals (CIs). Covariates were selected based on the change-in-estimate criterion (>10% HR alteration) or clinical relevance.

Adjustment models were constructed as follows: Crude: Unadjusted; Model 1: Adjusted for CA, HR, RR; Model 2: Model 1 plus Na, PLT, BUN; Model 3: Model 2 plus PT, APTT.

Supplementary analyses included: Nonlinearity: Assessed using restricted cubic splines (RCS, four knots); Survival: Analyzed with Kaplan–Meier curves and log-rank tests; Discrimination: Evaluated using receiver operating characteristic (ROC) curves and AUC comparisons. The software used for analysis was Free Statistical Software v2.2. Significance was determined at a two-sided *p*-value of < 0.05.

### 2.5 Subgroup and sensitivity analyses

Subgroup analyses were performed for the following variables: age (< 60/≥60), gender, coronary artery disease, cerebrovascular accident, diabetes mellitus, high blood pressure, bloodstream infection, and MAP (< 65/≥65 mmHg). Sensitivity analyses included: Exclusion of cases with bloodstream infection; Exclusion of non-CRRT patients to address confounding by CRRT.

## 3 Results

### 3.1 Study population and baseline characteristics

**Figure 1:**
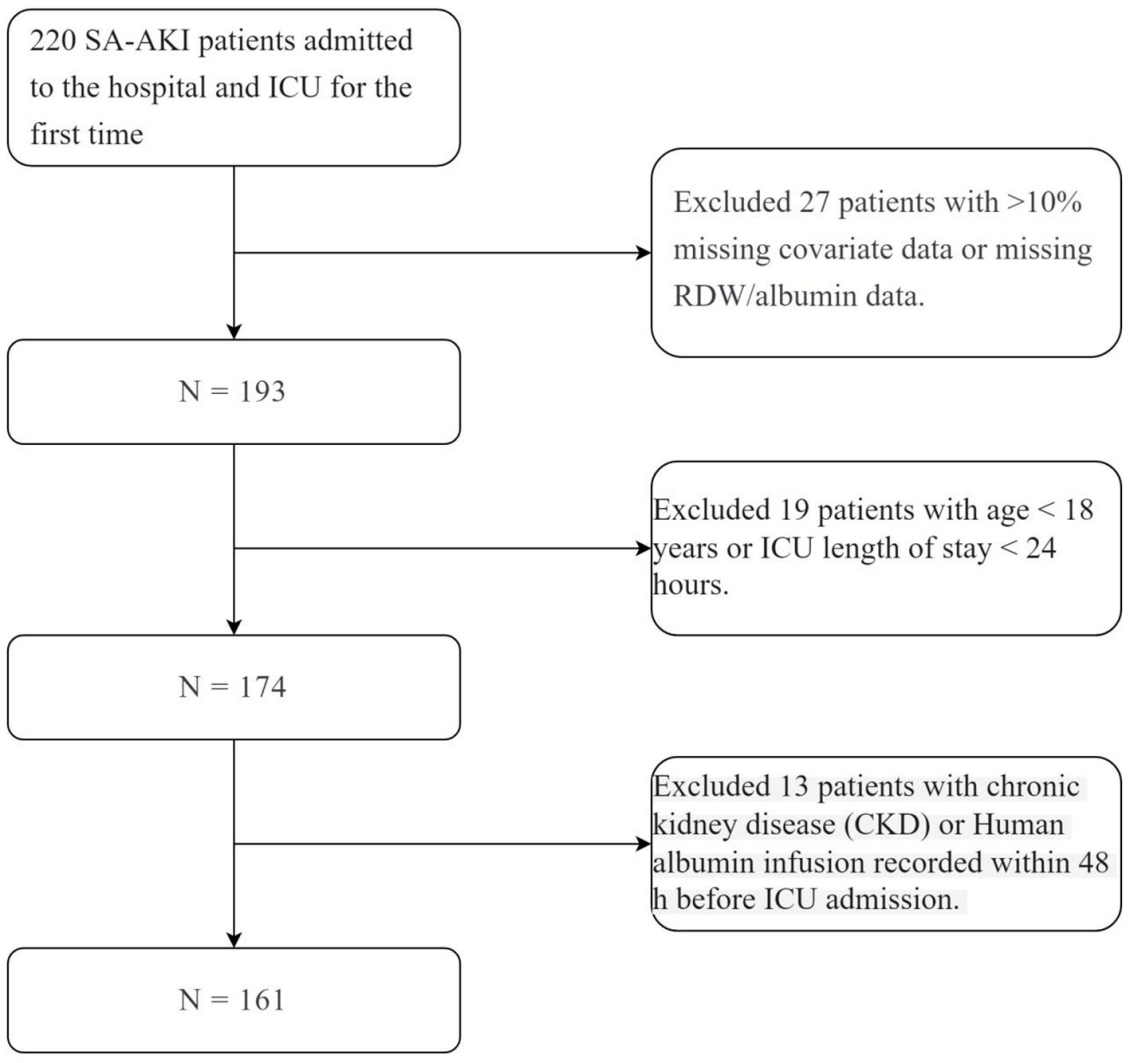
*N* = 161.

A total of 220 first-time SA-AKI patients, both first-time hospital and first-time ICU admissions, were screened. After applying exclusion criteria−27 patients with missing RDW/albumin data or more than 10% missing covariate data, 19 patients aged < 18 years or with ICU stays < 24 h, and 13 patients who received human albumin infusion within 48 h before ICU admission or had pre-existing chronic kidney disease−161 patients were ultimately included (Figure 1).

Baseline characteristics are summarized in Table 1: mean age was 63.7 years; 96 males (59.6%) and 65 females (40.4%); mean RAR was 6.95 ± 2.8 %/g/dl. The primary comorbidities were hypertension (39.8%), diabetes (30.4%), and cerebrovascular accident (24.8%). Stratification by RAR quartiles (Q1: < 5.07; Q2: 5.07–6.20; Q3: 6.20–8.31; Q4: >8.31) revealed that higher RAR groups exhibited: increased SOFA, APACHE II, lactate, LAR, and BNP; decreased hemoglobin, hematocrit, MAP, and albumin; and a higher incidence of mechanical ventilation use (all *p* < 0.05). Furthermore, there was an increased incidence of septic shock, vasopressor requirement, and CRRT utilization. The primary outcome, 28-day mortality, was 34.2% overall and increased progressively across RAR quartiles: 10% (RAR < 5.07), 32.5% (RAR 5.07–6.20), 42.5% (RAR 6.20–8.31), and 51.2% (RAR > 8.31). The inter-group comparison yielded a *p*-value < 0.001, indicating that higher RAR is associated with increased 28-day mortality in SA-AKI patients.

**Table 1:** Baseline clinical and laboratory characteristics of the study patients.

**Variables**	**RAR**					***p***
	**Total**	**Tertile 1**	**Tertile 2**	**Tertile 3**	**Tertile 4**	
		<**5.07**	**5.07–6.20**	**6.20–8.31**	>**8.31**	
	**(*****n*** = **161)**	**(*****n*** = **40)**	**(*****n*** = **40)**	**(*****n*** = **40)**	**(*****n*** = **41)**	
Age, mean ±*SD*	63.7 ± 16.6	66.8 ± 15.4	65.5 ± 15.1	64.7 ± 16.6	58.0 ± 18.0	0.071
**Gender**, ***n*** **(%)**						0.885
Male	96 (59.6)	22 (55)	25 (62.5)	25 (62.5)	24 (58.5)	
Female	65 (40.4)	18 (45)	15 (37.5)	15 (37.5)	17 (41.5)	
**CAD**, ***n*** **(%)**						0.105
No	141 (87.6)	33 (82.5)	34 (85)	34 (85)	40 (97.6)	
Yes	20 (12.4)	7 (17.5)	6 (15)	6 (15)	1 (2.4)	
**CA**, ***n*** **(%)**						0.688
No	152 (94.4)	37 (92.5)	37 (92.5)	38 (95)	40 (97.6)	
Yes	9 ( 5.6)	3 (7.5)	3 (7.5)	2 (5)	1 (2.4)	
**CVA**, ***n*** **(%)**						0.238
No	121 (75.2)	28 (70)	27 (67.5)	31 (77.5)	35 (85.4)	
Yes	40 (24.8)	12 (30)	13 (32.5)	9 (22.5)	6 (14.6)	
**DM**, ***n*** **(%)**						0.148
No	112 (69.6)	27 (67.5)	27 (67.5)	24 (60)	34 (82.9)	
Yes	49 (30.4)	13 (32.5)	13 (32.5)	16 (40)	7 (17.1)	
**HBP**, ***n*** **(%)**						0.215
No	97 (60.2)	22 (55)	26 (65)	20 (50)	29 (70.7)	
Yes	64 (39.8)	18 (45)	14 (35)	20 (50)	12 (29.3)	
**Vasoactive agents**, ***n*** **(%)**						< 0.001
No	43 (26.7)	21 (52.5)	8 (20)	9 (22.5)	5 (12.2)	
Yes	118 (73.3)	19 (47.5)	32 (80)	31 (77.5)	36 (87.8)	
**MV**, ***n*** **(%)**						0.001
No	40 (24.8)	18 (45)	12 (30)	5 (12.5)	5 (12.2)	
Yes	121 (75.2)	22 (55)	28 (70)	35 (87.5)	36 (87.8)	
**CRRT**, ***n*** **(%)**						< 0.001
No	99 (61.5)	33 (82.5)	27 (67.5)	23 (57.5)	16 (39)	
Yes	62 (38.5)	7 (17.5)	13 (32.5)	17 (42.5)	25 (61)	
HR, mean ±*SD*	117.8 ± 24.9	113.2 ± 24.9	116.9 ± 23.0	114.2 ± 21.7	126.6 ± 28.0	0.058
T, mean ±*SD*	38.0 ± 1.3	37.8 ± 1.3	38.2 ± 1.4	37.9 ± 1.2	38.1 ± 1.2	0.451
RR, mean ±*SD*	26.6 ± 9.1	24.1 ± 8.6	25.4 ± 8.7	27.9 ± 9.0	29.0 ± 9.8	0.067
MAP, mean ±*SD*	70.8 ± 18.5	80.4 ± 16.6	72.8 ± 17.3	70.2 ± 17.9	59.9 ± 16.6	< 0.001
APACHE II, mean ±*SD*	23.8 ± 8.3	20.1 ± 5.6	23.4 ± 7.8	24.4 ± 8.4	27.2 ± 9.6	0.001
SOFA, mean ±*SD*	9.5 ± 4.4	6.8 ± 3.0	9.8 ± 4.1	9.8 ± 4.3	11.8 ± 4.8	< 0.001
WBC, mean ±*SD*	17.3 ± 10.6	16.7 ± 7.8	20.2 ± 11.6	14.8 ± 9.1	17.3 ± 12.7	0.146
PLT, median (IQR)	139.0 (54.9, 229.0)	180.9 (131.3, 238.6)	127.3 (50.8, 207.5)	142.0 (60.0, 232.2)	68.0 (23.0, 159.0)	0.004
HGB, mean ±*SD*	96.8 ± 26.7	111.9 ± 22.8	101.2 ± 26.8	91.5 ± 23.1	83.0 ± 25.5	< 0.001
HCT, mean ±*SD*	34.1 ± 9.3	37.4 ± 8.4	35.2 ± 8.7	32.4 ± 8.3	31.3 ± 10.6	0.013
TBil, median (IQR)	17.8 (9.2, 37.1)	10.1 (7.2, 19.1)	22.4 (10.6, 51.5)	16.9 (9.0, 35.7)	28.5 (12.9, 51.1)	0.002
IBil, median (IQR)	5.1 (2.9, 9.7)	4.4 (2.9, 8.1)	5.2 (3.4, 9.8)	5.0 (2.9, 9.2)	6.2 (2.9, 12.6)	0.441
ALT, median (IQR)	31.0 (15.0, 106.0)	19.0 (15.0, 31.0)	91.0 (24.8, 205.8)	26.5 (14.8, 77.2)	56.0 (15.0, 130.0)	0.001
AST, median (IQR)	54.0 (26.0, 191.0)	28.5 (21.0, 49.0)	90.5 (41.0, 402.0)	53.0 (29.0, 93.2)	116.0 (37.0, 347.0)	< 0.001
ALB, mean ±*SD*	27.3 ± 6.6	34.3 ± 5.3	28.4 ± 5.0	24.2 ± 3.5	22.4 ± 5.2	< 0.001
Lac, median (IQR)	3.4 (2.0, 7.1)	2.6 (1.8, 5.0)	3.3 (1.7, 5.7)	3.1 (1.9, 5.7)	6.1 (3.1, 11.4)	< 0.001
${\text{HCO}}_{3}^{-}$, mean ±*SD*	18.3 ± 6.2	19.4 ± 6.8	20.0 ± 5.7	16.8 ± 6.2	16.9 ± 5.5	0.031
BUN, mean ±*SD*	15.2 ± 9.7	14.0 ± 12.2	15.3 ± 8.8	16.9 ± 9.6	14.6 ± 7.6	0.574
BNP, median (IQR)	3325.0 (1325.0, 10410.0)	2125.0 (420.4, 5412.5)	4499.0 (2010.8, 10449.8)	2247.0 (1159.8, 7401.8)	4458.0 (2320.0, 18838.0)	0.01
K, mean ±*SD*	4.3 ± 1.5	4.1 ± 1.0	4.0 ± 1.1	4.4 ± 1.3	4.8 ± 2.1	0.064
Na, mean ±*SD*	141.2 ±12.7	141.6 ± 13.0	140.3 ± 12.3	141.2 ± 14.4	141.5 ±11.4	0.97
PaO_2_/FiO_2_, mean ±*SD*	212.3 ± 103.3	238.5 ± 111.1	212.9 ± 83.1	190.7 ± 82.2	207.2 ± 127.0	0.219
CRP, median (IQR)	144.5 (79.6, 200.0)	109.0 (75.0, 203.2)	157.8 (97.3, 204.2)	147.8 (83.5, 200.0)	143.9 (88.6,200.0)	0.596
PCT, median (IQR)	11.0 (2.2, 87.2)	4.5 (1.6, 17.4)	24.8 (3.7, 81.5)	20.1 (2.7, 100.0)	12.9 (4.3, 90.8)	0.104
PT, median (IQR)	15.6 (13.4, 19.1)	13.9 (12.5, 17.0)	15.6 (13.9, 19.5)	15.2 (13.1, 17.8)	18.0 (14.6, 23.9)	0.002
APTT, median (IQR)	35.6 (30.8, 48.2)	32.0 (28.0, 35.8)	34.8 (31.2, 41.8)	36.7 (32.0, 45.2)	42.8 (36.0, 73.9)	< 0.001
D dimer, median (IQR)	2752.0 (1098.0, 5162.0)	2713.5 (1119.8, 3626.2)	3576.5 (1284.5, 7760.0)	2543.5 (806.0, 3957.0)	2742.0 (1234.0, 5637.0)	0.13
LAR, median (IQR)	1.4 (0.8,2.2)	0.8 (0.5, 1.4)	1.2 (0.7, 2.0)	1.4 (0.8, 2.3)	2.7 (1.4,5.3)	< 0.001
**Mortality 28 day**, ***n*** **(%)**						< 0.001
No	106 (65.8)	36 (90)	27 (67.5)	23 (57.5)	20 (48.8)	
Yes	55 (34.2)	4 (10)	13 (32.5)	17 (42.5)	21 (51.2)	

CAD, coronary artery disease; CA, cancer; CVA, cerebrovascular accident (stroke); DM, diabetes mellitus; HBP, high blood pressure; MV, mechanical ventilation; LAR, lactate-to- albumin ratio; T, body temperature; RR, respiratory rate; HR, heart rate; MAP, mean arterial pressure; SOFA, sequential organ failure assessment; APACHE II, acute physiology and chronic health evaluation II; WBC, white blood cell count; HCT, hematocrit; HGB, hemoglobin; PLT, platelet count; PCT, procalcitonin; RDW, red blood cell distribution width; RAR, RDW-to-albumin ratio; PT, prothrombin time; APTT, activated partial thromboplastin time; CRRT, continuous renal replacement therapy; IBil, indirect bilirubin.

### 3.2 Association between RAR and 28-day mortality

#### 3.2.1 Nonlinear analysis

**Figure 2:**
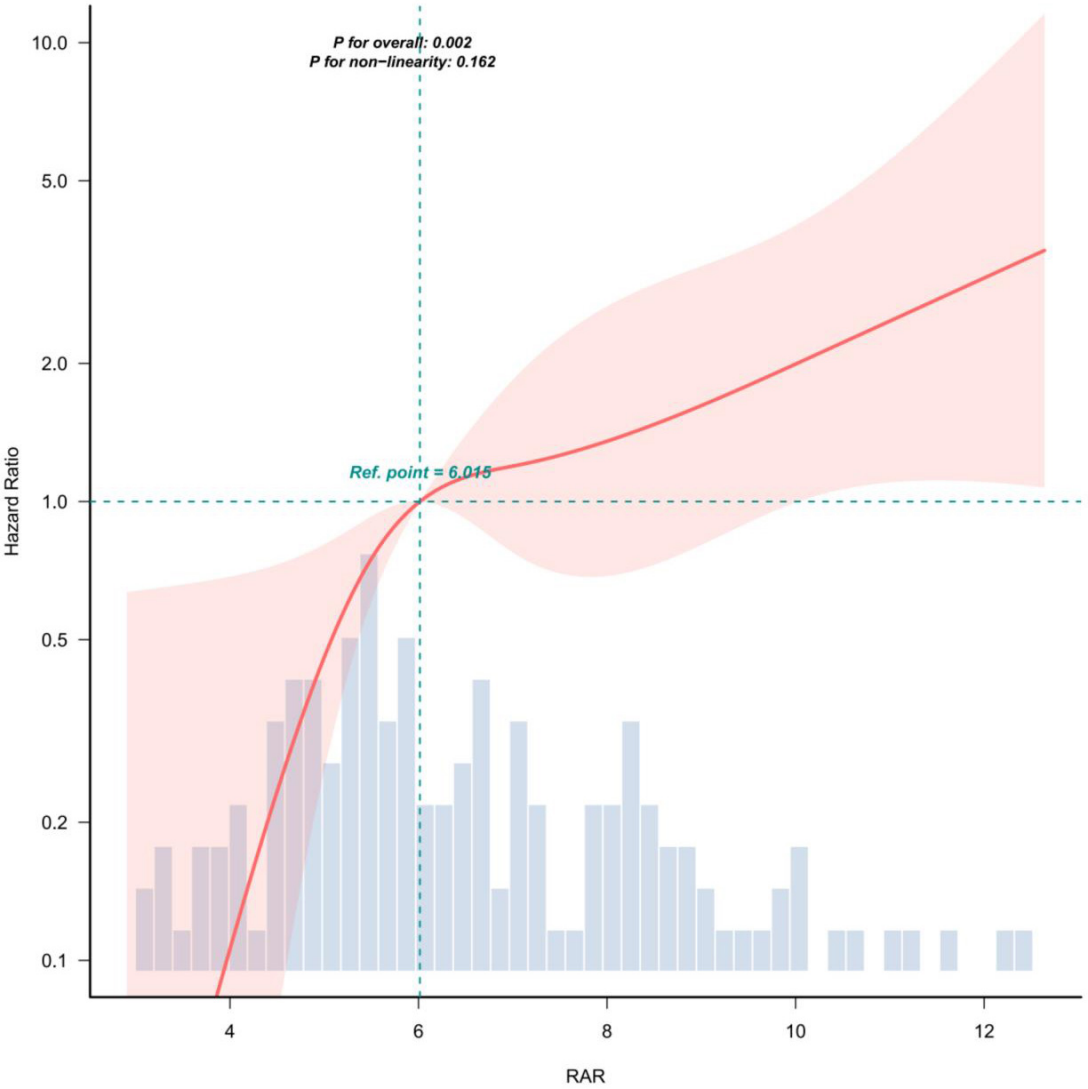
Covariate adjustments were consistent with model 3 in Table 3. The solid red line represents the estimated association, while the shaded red band indicates the corresponding 95% confidence interval (CI).

RCS analysis showed a linear relationship between RAR and mortality after covariate adjustment (non-linearity *p* = 0.162; Figure 2).

#### 3.2.2 Survival analysis

**Figure 3:**
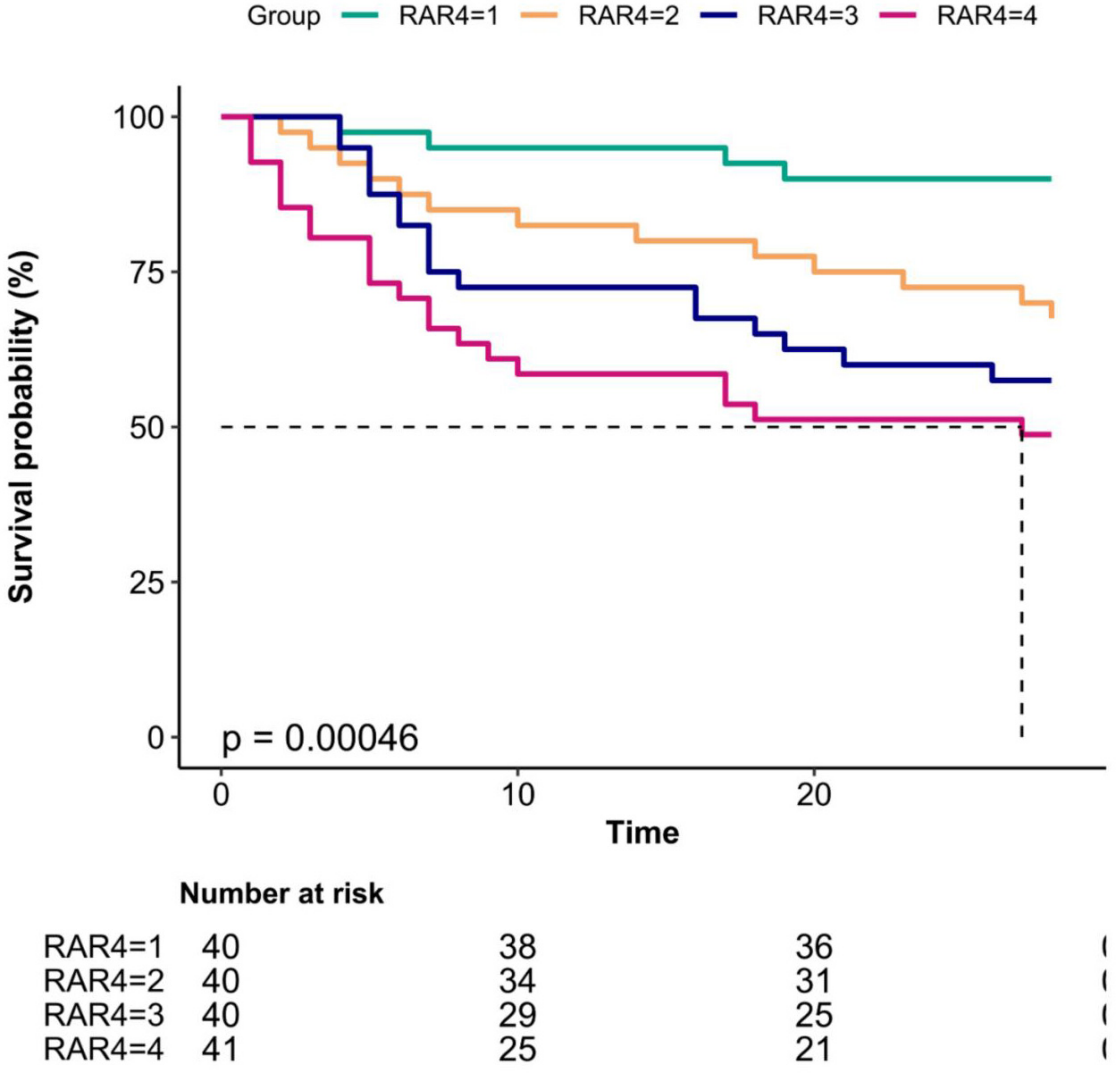
Kaplan–Meier curves for 28-day mortality in patients with sepsis-associated acute kidney injury (SA-AKI).

Kaplan–Meier curves revealed significantly higher 28-day survival rates in low-RAR groups (log-rank *p* < 0.001; Figure 3).

#### 3.2.3 Multivariable cox regression

After univariate screening (Table 2), progressively adjusted models demonstrated the following results: in the crude model, the HR for Q4 vs. Q1 was 7.28 (2.5–21.22; *p* < 0.05); in the fully adjusted model (Model 3), the HR for Q4 vs. Q1 was 7.52 (2.24–25.29; *p* < 0.05), adjusted for CA, HR, Na, PLT, PT, APTT, BUN, and RR (Table 3).

**Table 2:** Univariate cox regression hazard ratio of 28-day mortality associated with RAR in SA-AKI patients.

**Item**	**HR (95%CI)**	***p* (Wald's test)**
RAR (cont. var.)	1.11 (1.03, 1.19)	0.004
**RAR: ref**. = **Q1**		
Q2	3.63 (1.18, 11.12)	0.024
Q3	5.14 (1.73, 15.29)	0.003
Q4	7.28 (2.5, 21.22)	< 0.001
Age (cont. var.)	1.01 (1, 1.03)	0.101
Female vs. male	1.0002 (0.5831, 1.7154)	1
CAD: yes vs. no	1.54 (0.76, 3.15)	0.234
CA: yes vs. no	2.63 (1.12, 6.15)	0.026
CVA: yes vs. no	1.29 (0.72, 2.3)	0.397
DM: yes vs. no	1.01 (0.57, 1.79)	0.971
HBP: yes vs. no	1.14 (0.67, 1.95)	0.623
Vasoactive agents: yes vs. no	346499955 (0, Inf)	0.996
MV: yes vs. no	326653859.8 (0, Inf)	0.996
HR (cont. var.)	1.01 (1, 1.03)	0.016
TFR modify (cont. var.)	1.06 (0.86, 1.3)	0.575
RR (cont. var.)	1.04 (1.02, 1.07)	0.002
WBC (cont. var.)	0.9908 (0.9653, 1.017)	0.488
HGB (cont. var.)	0.9911 (0.9812, 1.0011)	0.08
TBil (cont. var.)	1.0035 (0.9984, 1.0086)	0.18
ALT (cont. var.)	1.0002 (0.9998, 1.0005)	0.388
AST (cont. var.)	1 (0.9999, 1.0002)	0.962
Na (cont. var.)	1.02 (1, 1.04)	0.032
PT (cont. var.)	1.01 (1, 1.02)	0.003
APTT (cont. var.)	1.0093 (1.0049, 1.0137)	< 0.001
HCT (cont. var.)	0.99 (0.96, 1.01)	0.328
PLT (cont. var.)	0.9959 (0.993, 0.9987)	0.005
BUN (cont. var.)	1.02 (1, 1.05)	0.033
${\text{HCO}}_{3}^{-}$ (cont. var.)	0.96 (0.92, 1.01)	0.1
CRP (cont. var.)	1.0002 (0.9979, 1.0024)	0.893
PCT (cont. var.)	1.0024 (0.9961, 1.0087)	0.457
D dimer (cont. var.)	1 (1, 1)	0.058

CAD, coronary artery disease; CA, cancer; CVA, cerebrovascular accident (stroke); DM, diabetes mellitus; HBP, high blood pressure; MV, mechanical ventilation; HCT, hematocrit; PLT, platelet count; RAR, RDW-to-albumin ratio; T, body temperature; RR, respiratory rate; HR, heart rate; PCT, procalcitonin; PT, prothrombin time; APTT, activated partial thromboplastin time.

**Table 3:** Hazard ratio of 28-day mortality in SA-AKI patients associated with RAR among septic patients.

**Variable**	**HR (95%CI)**								
	**No**.	**crude**	***p*** **-value**	**Model 1**	***p*** **-value**	**Model 2**	***p*** **-value**	**Model 3**	***p*** **-value**
RAR	161	1.11 (1.03–1.19)	0.004	1.1 (1.02–1.18)	0.012	1.11 (1.02–1.21)	0.016	1.1 (1–1.2)	0.04
**Quartiles**									
Q1 ( ≤ 5.07)	40	1 (Ref)		1 (Ref)		1 (Ref)		1 (Ref)	
Q2 (5.07–6.20)	40	3.63 (1.18–11.12)	0.024	4.1 (1.32–12.74)	0.015	3.68 (1.17–11.55)	0.026	4.6 (1.36–15.58)	0.014
Q3 (6.20–8.31)	40	5.14 (1.73–15.29)	0.003	5 (1.67–14.91)	0.004	4.4 (1.47–13.19)	0.008	5.38 (1.62–17.95)	0.006
Q4 (≥8.31)	41	7.28 (2.5–21.22)	< 0.001	6.79 (2.28–20.16)	0.001	6.01 (1.99–18.14)	0.001	7.52 (2.24–25.29)	0.001
Trend test	161		< 0.001		< 0.001		0.001		0.001

CA, cancer; HR, heart rate; RR, respiratory rate; PT, prothrombin time; APTT, activated partial thromboplastin time; PLT, platelet count; BUN, blood urea nitrogen.

Model 1: Adjusted for CA, HR, RR.

Model 2: Further adjusted for Na, PLT, BUN.

Model 3: Further adjusted for PT, APTT.

### 3.3 ROC curve analysis

RAR alone: AUC = 0.694 (95% CI: 0.612–0.776); RAR + LAR: AUC = 0.777 (95% CI: 0.703–0.851). The combined model significantly outperformed RAR alone (ΔAUC = 0.083, *p* = 0.043; Supplementary Figure S1).

### 3.4 Subgroup and sensitivity analyses

#### 3.4.1 Subgroup analysis

**Figure 4:**
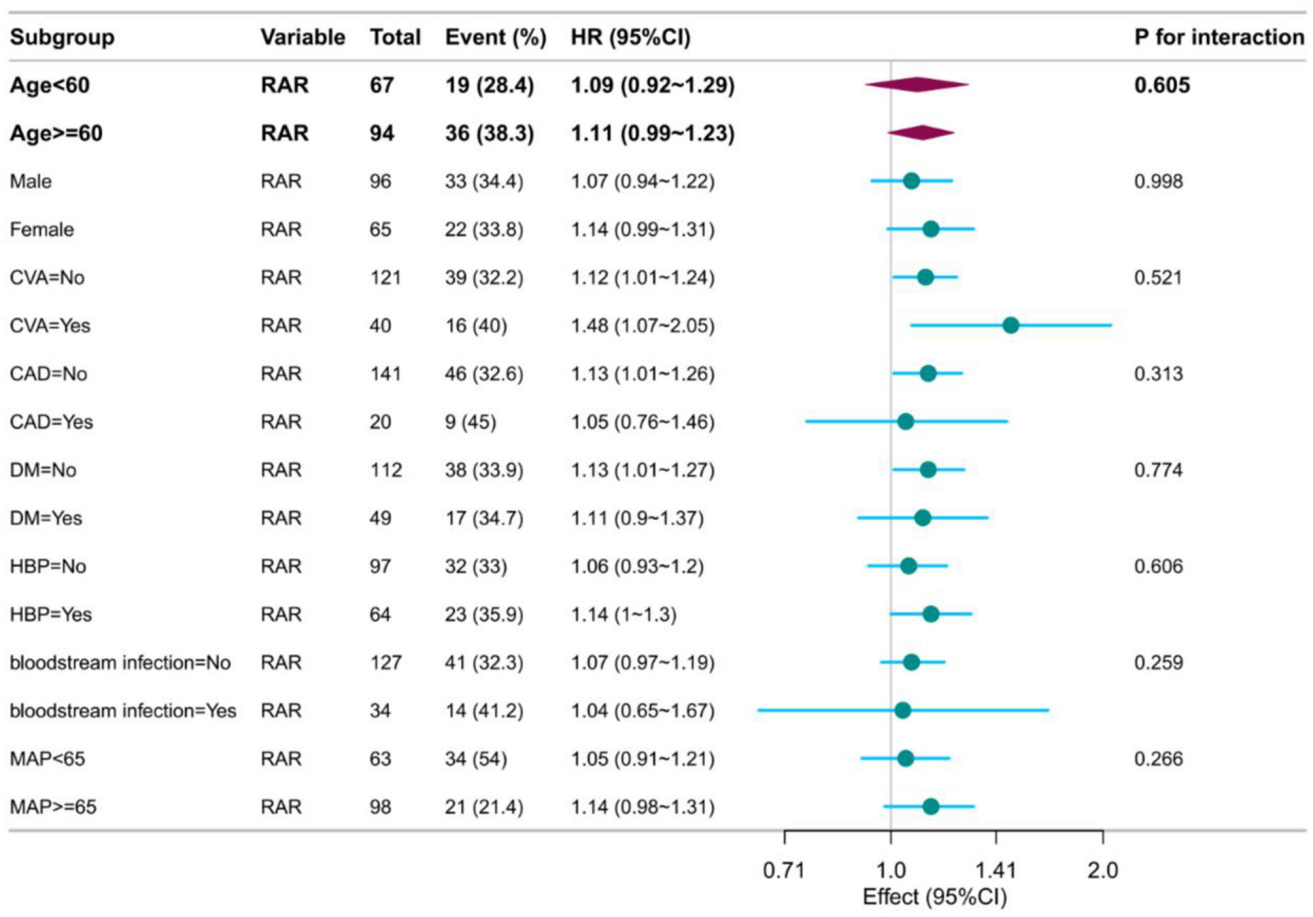
Forest plot of subgroup analysis.

No significant interaction effects were observed across subgroups (age, gender, coronary artery disease, stroke, diabetes, hypertension, bloodstream infection, MAP; all *p* for interaction >0.05; Figure 4).

#### 3.4.2 Sensitivity analyses

Excluding bloodstream infection patients: The association remained robust (Supplementary Table S1). Excluding non-CRRT patients: Results were consistent with the primary findings (Supplementary Table S2).

## 4 Discussion

This study is the first to evaluate the prognostic value of the RAR in SA-AKI. A retrospective analysis of 161 SA-AKI patients revealed three key findings: (1) Elevated RAR was independently associated with increased 28-day all-cause mortality (adjusted HR = 7.52, 95% CI: 2.24–25.29 for Q4 [RAR > 8.31] vs. Q1); (2) RAR correlated positively with disease severity, with higher RAR groups showing significantly increased SOFA scores (11.8 vs. 6.8), APACHE II scores (27.2 vs. 20.1), mechanical ventilation rates (87.8% vs. 55%), and CRRT utilization (61% vs. 17.5%; all *p* < 0.05); (3) Combining RAR with the lactate-to-albumin ratio (LAR) significantly improved predictive accuracy (AUC = 0.777 vs. 0.694 for RAR alone; ΔAUC = 0.083, *p* = 0.043.

### 4.1 Novelty and pathophysiological insights

This study positions RAR as a novel independent prognostic marker for SA-AKI, addressing a critical knowledge gap in sepsis-induced renal injury—an area previously focused on conditions such as diabetic ketoacidosis (13), cancer (15), aortic aneurysm (19), and ARDS (20). Its innovation lies in integrating two key pathophysiological mechanisms of sepsis: (1) Elevated RDW reflects erythropoietic dysfunction: During sepsis, systemic infection and inflammation frequently impair hematopoiesis by suppressing erythropoietin production and disrupting red-cell maturation, which increases the proportion of immature erythrocytes in circulation (21). Inflammatory mediators reduce iron availability and accelerate erythrocyte apoptosis, contributing to sepsis-associated anemia (22, 23). Cytokines also alter erythrocyte membrane glycoproteins and ion channels, inducing morphological changes and compromising membrane stability (24, 25). This results in the release of immature, heterogeneous-sized red cells, explaining RDW's prognostic value in sepsis (8). (2) Hypoalbuminemia signals microcirculatory failure: Physiologically, serum albumin acts as an extracellular antioxidant, buffer, immune modulator, detoxifier, and transport protein. Its decline reflects the intensity of inflammation, oxidative stress, and capillary leak. Albumin modulates organ perfusion by inhibiting platelet activation (26, 27) and preserving endothelial integrity (28); lower levels correlate with worse AKI outcomes (29). (3) The integration of RAR (RDW/Alb) amplifies the pathological signal: RAR concurrently captures impaired erythropoiesis and hypoalbuminemia. The ratio of RDW (reflecting oxidative stress and marrow suppression) to albumin encapsulates the interplay between inflammation, microcirculatory compromise, and malnutrition (30, 31). When hematopoietic suppression (↑RDW) coexists with capillary leakage (↓Alb), RAR rises exponentially, becoming a sensitive indicator of combined “inflammatory-microcirculatory” derangement.

### 4.2 Clinical implications and comparative value

Complementarity to conventional scores: While SOFA and APACHE II effectively predict SA-AKI outcomes (5, 6), their multi-parameter nature limits clinical utility. This study demonstrates that RAR is a readily obtainable, independent prognosticator. Patients in the Q4 group (RAR > 8.31) required significantly more vasopressors (87.8% vs. 47.5%) and CRRT (61% vs. 17.5%), highlighting RAR's potential as an early-warning marker for treatment escalation.

Synergistic predictive model: The biological basis of the LAR can be explained from two perspectives. Lactate, the end product of anaerobic metabolism, directly reflects tissue hypoperfusion and cellular hypoxia (32, 33), with the systemic inflammatory response syndrome (SIRS) and microcirculatory failure induced by sepsis driving lactate elevation. Serum albumin serves as an extracellular antioxidant, buffer, immune modulator, detoxifier, and transport protein, and its decline parallels the intensity of inflammation, oxidative stress, and capillary leakage (34, 35). Therefore, LAR integrates the dual pathological signals of tissue hypoxia/under-perfusion (lactate) and antioxidant/immune-endothelial integrity loss (albumin), encapsulating the “ischaemia–reperfusion injury–SIRS” axis.

RAR + LAR achieves multi-dimensional coverage: RAR reflects erythrocyte damage (RDW) and inflammation (Alb), while LAR captures tissue hypoxia (Lac) and oxidative stress (Alb) (36). Together, they comprehensively encompass the central mechanisms driving SA-AKI progression, including systemic inflammation, microcirculatory collapse, and multiple organ dysfunction. The combined AUC of 0.777 (95% CI: 0.703–0.851) significantly outperformed RAR alone (*p* = 0.043).

Previous studies reported AUCs for SA-AKI mortality: 0.624 for neutrophil-lymphocyte ratio (NLR), 0.582 for platelet-lymphocyte ratio, 0.599 for monocyte-lymphocyte ratio, 0.590 for systemic immune-inflammation index, and 0.593 for systemic inflammatory response index (37). Serum cystatin C yielded an AUC of 0.690 (38). Both RAR and the RAR-LAR combination outperform these traditional indices, laying the groundwork for a simple yet robust prognostic tool in SA-AKI.

### 4.3 Generalizability and future directions

Prespecified subgroup analyses (age, gender, diabetes, coronary artery disease, bloodstream infection, etc.) confirmed consistent prognostic associations (all interaction *p* > 0.05). Notably, predictive power remained in high-risk subgroups, including diabetes (*n* = 49, HR = 1.11) and hypertension (*n* = 64, HR = 1.14), highlighting the robustness of RAR against potential confounding.

Future research directions: (1) Dynamic trajectory analysis: Investigating whether changes in RAR during treatment outperform baseline values; (2) Mechanistic validation: Animal models to explore RAR's causal links to renal medullary hypoxia and ferroptosis.

### 4.4 Limitations and mitigation strategies

(1) Single-center retrospective design: Despite rigorous multivariable adjustment, the findings require multicenter prospective validation, ideally across diverse ICU populations; (2) Sample size constraints (*n* = 161): Limited power for rare subgroups (e.g., malignancy cohort, *n* = 9); (3) Lack of serial measurements: An expanded cohort (target *N* = 500) is underway to track RAR dynamics over time.

## 5 Conclusion

Elevated RAR independently predicts adverse early prognosis in SA-AKI, with higher levels correlating with increased 28-day mortality. The combination of RAR and LAR significantly improves mortality prediction in this cohort.

## Data Availability

The raw data supporting the conclusions of this article will be made available by the authors, without undue reservation.
