# Supplementary material for: Association between red blood cell distribution width to albumin ratio and prognosis in patients with sepsis-associated acute kidney injury: a retrospective cohort study
Source: Front Med (Lausanne). 2026 Feb 3;13:1724095. doi: 10.3389/fmed.2026.1724095 (PMC12909163; doi:10.3389/fmed.2026.1724095)
Supplement: Supplementary file 1 [file Data_Sheet_1.docx]

**Table S1. Hazard Ratio of 28-Day Mortality for SA-AKI Patients Associated with RAR in Septic Patients after Excluding Those with Concomitant Bloodstream Infection.**

| RAR | HR(95%CI) | | | | |
| --- | --- | --- | --- | --- | --- |
|  | NO. | crude | *P* value | Model 3 | *P* value |
| Quartiles |  |  |  |  |  |
| Q1 | 32 | 1(Ref) |  | 1(Ref) |  |
| Q2 | 35 | 3.26 (0.9~11.86) | 0.072 | 4.49 (1.08~18.71) | 0.039 |
| Q3 | 29 | 5.96 (1.7~20.95) | 0.005 | 6.65 (1.63~27.18) | 0.008 |
| Q4 | 31 | 6.98 (2.02~24.12) | 0.002 | 7.63 (1.83~31.8) | 0.005 |
| Trend.test | 127 |  | <0.001 |  | 0.003 |

**Abbreviations:** CA, cancer; HR, Heart rate; RR, Respiratory rate; PT, Prothrombin time; APTT, Activated partial thromboplastin time; PLT, Platelet count; BUN, Blood Urea Nitrogen.

**Model 3:** Adjusted for Adjusted for CA, HR, Na, PLT, PT, APTT, BUN, and RR.
