# Supplementary material for: Association between red blood cell distribution width to albumin ratio and prognosis in patients with sepsis-associated acute kidney injury: a retrospective cohort study
Source: Front Med (Lausanne). 2026 Feb 3;13:1724095. doi: 10.3389/fmed.2026.1724095 (PMC12909163; doi:10.3389/fmed.2026.1724095)
Supplement: Supplementary file 2 [file Data_Sheet_2.docx]

**Table S2. Hazard Ratio of 28-Day Mortality for SA-AKI Patients Associated with RAR in Septic Patients after Excluding Those Who Did Not Receive CRRT.**

| RAR | HR(95%CI) | | | | |
| --- | --- | --- | --- | --- | --- |
|  | NO. | crude | *P* value | Model 3 | *P* value |
| Quartiles |  |  |  |  |  |
| Q1 | 7 | 1(Ref) |  | 1(Ref) |  |
| Q2 | 13 | 5.1 (0.64~40.84) | 0.125 | 561779.81 (1.08~292308575713.67) | 0.049 |
| Q3 | 17 | 5.68 (0.73~44.11) | 0.097 | 483177.58 (1.26~185941548679.07) | 0.046 |
| Q4 | 25 | 7.01 (0.93~52.94) | 0.059 | 628242.17 (1.61~245375782308.89) | 0.042 |
| Trend.test | 62 |  | 0.047 |  | 0.036 |

**Abbreviations:** CA, cancer; HR, Heart rate; RR, Respiratory rate; PT, Prothrombin time; APTT, Activated partial thromboplastin time; PLT, Platelet count; BUN, Blood Urea Nitrogen.

**Model 3:** Adjusted for Adjusted for CA, HR, Na, PLT, PT, APTT, BUN, and RR.
