# Supplementary material for: Association between red blood cell distribution width to albumin ratio and prognosis in patients with sepsis-associated acute kidney injury: a retrospective cohort study
Source: Front Med (Lausanne). 2026 Feb 3;13:1724095. doi: 10.3389/fmed.2026.1724095 (PMC12909163; doi:10.3389/fmed.2026.1724095)

**Figure S1. Receiver operating characteristic curve for predicting 28-day mortality in SA-AKI patients.**


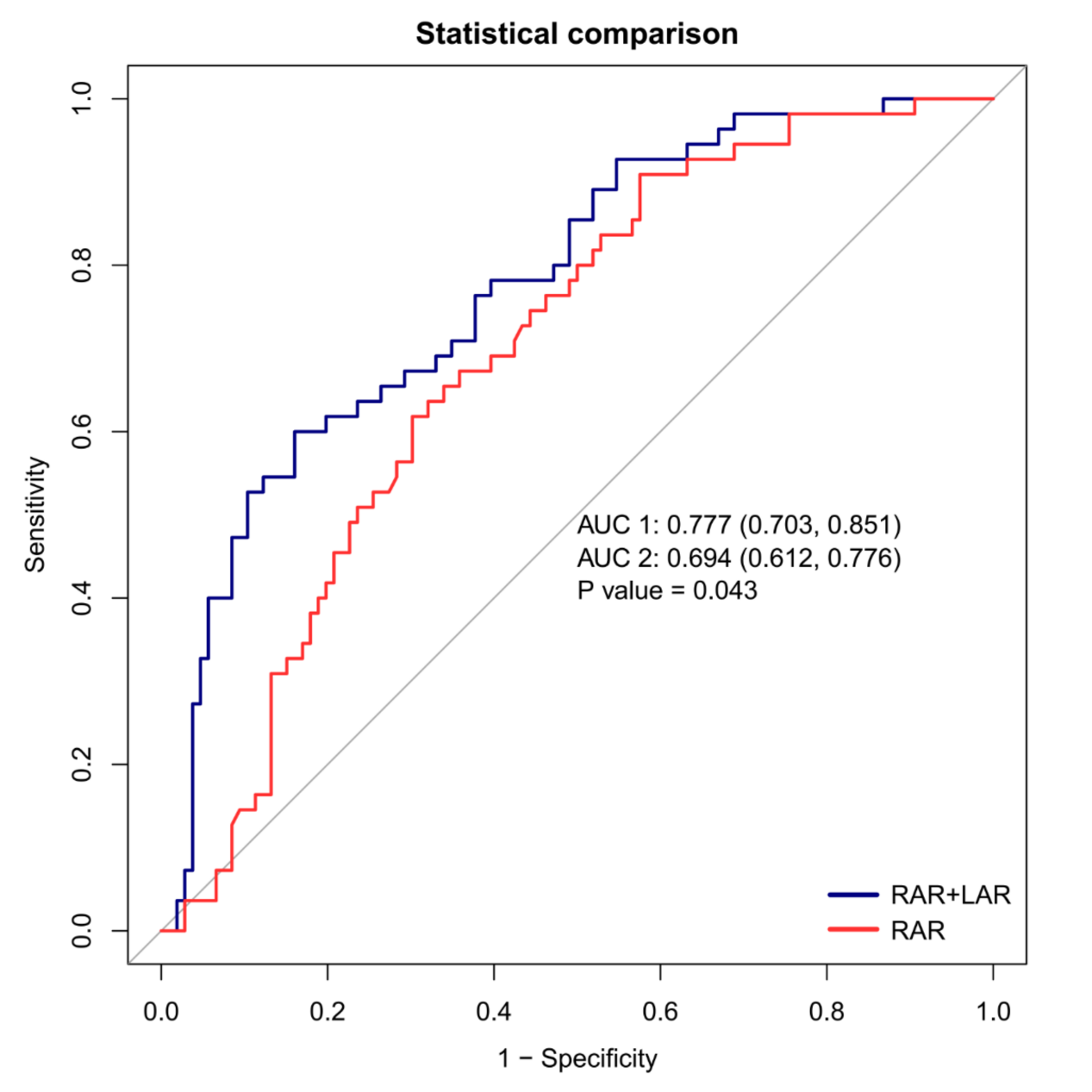

Supplement: Supplementary file 3 [file Data_Sheet_3.docx]
